# Bullying at school and mental health problems among adolescents: a repeated cross-sectional study

**DOI:** 10.1186/s13034-021-00425-y

**Published:** 2021-12-14

**Authors:** Håkan Källmén, Mats Hallgren

**Affiliations:** 1 Stockholm Prevents Alcohol and Drug Problems (STAD), Center for Addiction Research and Department of Clinical Neuroscience, Karolinska Institutet, Solna, Sweden; 2 Epidemiology of Psychiatric Conditions, Substance Use and Social Environment (EPiCSS), Department of Global Public Health, Karolinska Institutet, Level 6, Solnavägen 1e, Solna, Sweden

**Keywords:** Bullying, Mental health, Adolescents, School-related factors, Gender differences

## Abstract

**Objective:**

To examine recent trends in bullying and mental health problems among adolescents and the association between them.

**Method:**

A questionnaire measuring mental health problems, bullying at school, socio-economic status, and the school environment was distributed to all secondary school students aged 15 (school-year 9) and 18 (school-year 11) in Stockholm during 2014, 2018, and 2020 (n = 32,722). Associations between bullying and mental health problems were assessed using logistic regression analyses adjusting for relevant demographic, socio-economic, and school-related factors.

**Results:**

The prevalence of bullying remained stable and was highest among girls in year 9; range = 4.9% to 16.9%. Mental health problems increased; range = + 1.2% (year 9 boys) to + 4.6% (year 11 girls) and were consistently higher among girls (17.2% in year 11, 2020). In adjusted models, having been bullied was detrimentally associated with mental health (OR = 2.57 [2.24–2.96]). Reports of mental health problems were four times higher among boys who had been bullied compared to those not bullied. The corresponding figure for girls was 2.4 times higher.

**Conclusions:**

Exposure to bullying at school was associated with higher odds of mental health problems. Boys appear to be more vulnerable to the deleterious effects of bullying than girls.

**Supplementary Information:**

The online version contains supplementary material available at 10.1186/s13034-021-00425-y.

## Introduction

Bullying involves repeated hurtful actions between peers where an imbalance of power exists [1]. Arseneault et al. [2] conducted a review of the mental health consequences of bullying for children and adolescents and found that bullying is associated with severe symptoms of mental health problems, including self-harm and suicidality. Bullying was shown to have detrimental effects that persist into late adolescence and contribute independently to mental health problems. Updated reviews have presented evidence indicating that bullying is causative of mental illness in many adolescents [3, 4].

There are indications that mental health problems are increasing among adolescents in some Nordic countries. Hagquist et al. [5] examined trends in mental health among Scandinavian adolescents (n = 116, 531) aged 11–15 years between 1993 and 2014. Mental health problems were operationalized as difficulty concentrating, sleep disorders, headache, stomach pain, feeling tense, sad and/or dizzy. The study revealed increasing rates of adolescent mental health problems in all four counties (Finland, Sweden, Norway, and Denmark), with Sweden experiencing the sharpest increase among older adolescents, particularly girls. Worsening adolescent mental health has also been reported in the United Kingdom. A study of 28,100 school-aged adolescents in England found that two out of five young people scored above thresholds for emotional problems, conduct problems or hyperactivity [6]. Female gender, deprivation, high needs status (educational/social), ethnic background, and older age were all associated with higher odds of experiencing mental health difficulties.

Bullying is shown to increase the risk of poor mental health and may partly explain these detrimental changes. Le et al. [7] reported an inverse association between bullying and mental health among 11–16-year-olds in Vietnam. They also found that poor mental health can make some children and adolescents more vulnerable to bullying at school. Bayer et al. [8] examined links between bullying at school and mental health among 8–9-year-old children in Australia. Those who experienced bullying more than once a week had poorer mental health than children who experienced bullying less frequently. Friendships moderated this association, such that children with more friends experienced fewer mental health problems (protective effect). Hysing et al. [9] investigated the association between experiences of bullying (as a victim or perpetrator) and mental health, sleep disorders, and school performance among 16–19 year olds from Norway (n = 10,200). Participants were categorized as victims, bullies, or bully-victims (that is, victims who also bullied others). All three categories were associated with worse mental health, school performance, and sleeping difficulties. Those who had been bullied also reported more emotional problems, while those who bullied others reported more conduct disorders [9].

As most adolescents spend a considerable amount of time at school, the school environment has been a major focus of mental health research [10, 11]. In a recent review, Saminathen et al. [12] concluded that school is a potential protective factor against mental health problems, as it provides a socially supportive context and prepares students for higher education and employment. However, it may also be the primary setting for protracted bullying and stress [13]. Another factor associated with adolescent mental health is parental socio-economic status (SES) [14]. A systematic review indicated that lower parental SES is associated with poorer adolescent mental health [15]. However, no previous studies have examined whether SES modifies or attenuates the association between bullying and mental health. Similarly, it remains unclear whether school related factors, such as school grades and the school environment, influence the relationship between bullying and mental health. This information could help to identify those adolescents most at risk of harm from bullying.

To address these issues, we investigated the prevalence of bullying at school and mental health problems among Swedish adolescents aged 15–18 years between 2014 and 2020 using a population-based school survey. We also examined associations between bullying at school and mental health problems adjusting for relevant demographic, socioeconomic, and school-related factors. We hypothesized that: (1) bullying and adolescent mental health problems have increased over time; (2) There is an association between bullying victimization and mental health, so that mental health problems are more prevalent among those who have been victims of bullying; and (3) that school-related factors would attenuate the association between bullying and mental health.

## Method

### Participants

The Stockholm school survey is completed every other year by students in lower secondary school (year 9—compulsory) and upper secondary school (year 11). The survey is mandatory for public schools, but voluntary for private schools. The purpose of the survey is to help inform decision making by local authorities that will ultimately improve students’ wellbeing. The questions relate to life circumstances, including SES, schoolwork, bullying, drug use, health, and crime. Non-completers are those who were absent from school when the survey was completed (< 5%). Response rates vary from year to year but are typically around 75%. For the current study data were available for 2014, 2018 and 2020. In 2014; 5235 boys and 5761 girls responded, in 2018; 5017 boys and 5211 girls responded, and in 2020; 5633 boys and 5865 girls responded (total n = 32,722). Data for the exposure variable, bullied at school, were missing for 4159 students, leaving 28,563 participants in the crude model. The fully adjusted model (described below) included 15,985 participants. The mean age in grade 9 was 15.3 years (SD = 0.51) and in grade 11, 17.3 years (SD = 0.61). As the data are completely anonymous, the study was exempt from ethical approval according to an earlier decision from the Ethical Review Board in Stockholm (2010-241 31-5). Details of the survey are available via a website [16], and are described in a previous paper [17].

### Procedure

Students completed the questionnaire during a school lesson, placed it in a sealed envelope and handed it to their teacher. Student were permitted the entire lesson (about 40 min) to complete the questionnaire and were informed that participation was voluntary (and that they were free to cancel their participation at any time without consequences). Students were also informed that the Origo Group was responsible for collection of the data on behalf of the City of Stockholm.

### Study outcome

*Mental health problems* were assessed by using a modified version of the Psychosomatic Problem Scale [18] shown to be appropriate for children and adolescents and invariant across gender and years. The scale was later modified [19]. In the modified version, items about difficulty concentrating and feeling giddy were deleted and an item about ‘life being great to live’ was added. Seven different symptoms or problems, such as headaches, depression, feeling fear, stomach problems, difficulty sleeping, believing it’s great to live (coded negatively as seldom or rarely) and poor appetite were used. Students who responded (on a 5-point scale) that any of these problems typically occurs ‘at least once a week’ were considered as having indicators of a mental health problem. Cronbach alpha was 0.69 across the whole sample. Adding these problem areas, a total index was created from 0 to 7 mental health symptoms. Those who scored between 0 and 4 points on the total symptoms index were considered to have a low indication of mental health problems (coded as 0); those who scored between 5 and 7 symptoms were considered as likely having mental health problems (coded as 1).

### Primary exposure

*Experiences of bullying* were measured by the following two questions: Have you felt bullied or harassed during the past school year? Have you been involved in bullying or harassing other students during this school year? Alternatives for the first question were: yes or no with several options describing how the bullying had taken place (if yes). Alternatives indicating emotional bullying were feelings of being mocked, ridiculed, socially excluded, or teased. Alternatives indicating physical bullying were being beaten, kicked, forced to do something against their will, robbed, or locked away somewhere. The response alternatives for the second question gave an estimation of how often the respondent had participated in bullying others (from once to several times a week). Combining the answers to these two questions, five different categories of bullying were identified: (1) never been bullied and never bully others; (2) victims of emotional (verbal) bullying who have never bullied others; (3) victims of physical bullying who have never bullied others; (4) victims of bullying who have also bullied others; and (5) perpetrators of bullying, but not victims. As the number of positive cases in the last three categories was low (range = 3–15 cases) bully categories 2–4 were combined into one primary exposure variable: ‘bullied at school’.

### Covariates

*Assessment year* was operationalized as the year when data was collected: 2014, 2018, and 2020. *Age* was operationalized as school grade 9 (15–16 years) or 11 (17–18 years). *Gender* was self-reported (boy or girl). *The school situation* To assess experiences of the school situation, students responded to 18 statements about well-being in school, participation in important school matters, perceptions of their teachers, and teaching quality. Responses were given on a four-point Likert scale ranging from ‘do not agree at all’ to ‘fully agree’. To reduce the 18-items down to their essential factors, we performed a principal axis factor analysis. Results showed that the 18 statements formed five factors which, according to the Kaiser criterion (eigen values > 1) explained 56% of the covariance in the student’s experience of the school situation. The five factors identified were: (1) Participation in school; (2) Interesting and meaningful work; (3) Feeling well at school; (4) Structured school lessons; and (5) Praise for achievements. For each factor, an index was created that was dichotomised (poor versus good circumstance) using the median-split and dummy coded with ‘good circumstance’ as reference. A description of the items included in each factor is available as Additional file 1. *Socio-economic status* (SES) was assessed with three questions about the education level of the student’s mother and father (dichotomized as university degree versus not), and the amount of spending money the student typically received for entertainment each month (> SEK 1000 [approximately $120] versus less). Higher parental education and more spending money were used as reference categories. *School grades* in Swedish, English, and mathematics were measured separately on a 7-point scale and dichotomized as high (grades A, B, and C) versus low (grades D, E, and F). High school grades were used as the reference category.

### Statistical analyses

The prevalence of mental health problems and bullying at school are presented using descriptive statistics, stratified by survey year (2014, 2018, 2020), gender, and school year (9 versus 11). As noted, we reduced the 18-item questionnaire assessing school function down to five essential factors by conducting a principal axis factor analysis (see Additional file 1). We then calculated the association between bullying at school (defined above) and mental health problems using multivariable logistic regression. Results are presented as odds ratios (OR) with 95% confidence intervals (Cis). To assess the contribution of SES and school-related factors to this association, three models are presented: Crude, Model 1 adjusted for demographic factors: age, gender, and assessment year; Model 2 adjusted for Model 1 plus SES (parental education and student spending money), and Model 3 adjusted for Model 2 plus school-related factors (school grades and the five factors identified in the principal factor analysis). These covariates were entered into the regression models in three blocks, where the final model represents the fully adjusted analyses. In all models, the category ‘not bullied at school’ was used as the reference. Pseudo R-square was calculated to estimate what proportion of the variance in mental health problems was explained by each model. Unlike the R-square statistic derived from linear regression, the Pseudo R-square statistic derived from logistic regression gives an indicator of the explained variance, as opposed to an exact estimate, and is considered informative in identifying the relative contribution of each model to the outcome [20]. All analyses were performed using SPSS v. 26.0.

## Results

### Prevalence of bullying at school and mental health problems

Estimates of the prevalence of bullying at school and mental health problems across the 12 strata of data (3 years × 2 school grades × 2 genders) are shown in Table 1. The prevalence of bullying at school increased minimally (< 1%) between 2014 and 2020, except among girls in grade 11 (2.5% increase). Mental health problems increased between 2014 and 2020 (range = 1.2% [boys in year 11] to 4.6% [girls in year 11]); were three to four times more prevalent among girls (range = 11.6% to 17.2%) compared to boys (range = 2.6% to 4.9%); and were more prevalent among older adolescents compared to younger adolescents (range = 1% to 3.1% higher). Pooling all data, reports of mental health problems were four times more prevalent among boys who had been victims of bullying compared to those who reported no experiences with bullying. The corresponding figure for girls was two and a half times as prevalent.

**Table 1:** Number of adolescents reporting mental health problems and experiences of bullying at school, stratified by year, school grade, and gender (n = 28,563)

Year	Grade	Gender	Sample n	Mental health problem/bullied at school	Number of cases	%	% change 2014–2020
2014	9	Boys	2558	MHP	64	2.6	+2.2
				Bullied	186	7.3	+0.7
		Girls	2481	MHP	283	11.6	+3.6
				Bullied	403	16.2	+0.7
	11	Boys	2673	MHP	93	3.5	+1.2
				Bullied	114	4.3	+0.6
		Girls	3280	MHP	406	12.6	+4.6
				Bullied	225	7.8	+2.5
2018	9	Boys	2652	MHP	90	3.5	–
				Bullied	209	7.9	–
		Girls	2595	MHP	356	14.0	–
				Bullied	391	15.1	–
	11	Boys	2365	MHP	112	4.9	–
				Bullied	115	4.9	–
		Girls	2616	MHP	441	17.1	–
				Bullied	307	11.7	–
2020	9	Boys	3176	MHP	132	4.4	–
				Bullied	253	8.0	–
		Girls	3094	MHP	465	15.2	–
				Bullied	523	16.9	–
	11	Boys	2457	MHP	112	4.7	–
				Bullied	121	4.9	–
		Girls	2771	MHP	468	17.2	–
				Bullied	285	10.3	–

*MHP* mental health problem, *Bullied* bullied at school

### Associations between bullying at school and mental health problems

Table 2 shows the association between bullying at school and mental health problems after adjustment for relevant covariates. Demographic factors, including female gender (OR = 3.87; CI 3.48–4.29), older age (OR = 1.38, CI 1.26–1.50), and more recent assessment year (OR = 1.18, CI 1.13–1.25) were associated with higher odds of mental health problems. In Model 2, none of the included SES variables (parental education and student spending money) were associated with mental health problems. In Model 3 (fully adjusted), the following school-related factors were associated with higher odds of mental health problems: lower grades in Swedish (OR = 1.42, CI 1.22–1.67); uninteresting or meaningless schoolwork (OR = 2.44, CI 2.13–2.78); feeling unwell at school (OR = 1.64, CI 1.34–1.85); unstructured school lessons (OR = 1.31, CI = 1.16–1.47); and no praise for achievements (OR = 1.19, CI 1.06–1.34). After adjustment for all covariates, being bullied at school remained associated with higher odds of mental health problems (OR = 2.57; CI 2.24–2.96). Demographic and school-related factors explained 12% and 6% of the variance in mental health problems, respectively (Pseudo R-Square). The inclusion of socioeconomic factors did not alter the variance explained.

**Table 2:** Association between bullying at school and mental health problems adjusted for demographic, socioeconomic, and school-related factors

	Crude		Model 1		Model 2		Model 3	
	OR	95% CI	OR	95% CI	OR	95% CI	OR	95% CI
Bullied at school (Crude, n = 28,563)	**3.73**	**3.40–4.10**	**3.28**	**2.98–3.64**	**3.28**	**2.89–3.72**	**2.57**	**2.24–2.96**
Demographic factors (Model 1, n = 27,614)								
Age (ref = grade 9)			**1.38**	**1.26–1.50**	**1.39**	**1.24–1.55**	**1.48**	**1.30–1.68**
Gender (ref = boys)			**3.87**	**3.48–4.29**	**4.33**	**3.78–4.96**	**4.42**	**3.80–5.13**
Assessment year (ref = 2014)			**1.18**	**1.13–1.25**	**1.22**	**1.14–1.30**	**1.23**	**1.14–1.32**
Socioeconomic factors (Model 2, n = 18,712)								
Mother university education (ref = yes)					1.00	0.87–1.14	0.96	0.83–1.11
Father university education (ref = yes)					1.22	1.08–1.39	1.14	0.99–1.30
Monthly spending money (ref = high)					1.05	0.94–1.16	1.06	0.94–1.16
School-related factors (Model 3, n = 15,985)								
Grade in Swedish (ref = high)							**1.42**	**1.22–1.67**
Grade in English (ref = high)							**0.79**	**0.67–0.95**
Grade in Mathematics (ref = high)							1.09	0.96–1.23
Participation in school (ref = high)							1.11	0.97–1.28
Interesting/meaningful schoolwork (ref = yes)							**2.44**	**2.13–2.78**
Feel well at school (ref = yes)							**1.64**	**1.34–1.85**
School lessons structured (ref = yes)							**1.31**	**1.16–1.47**
Praise for achievements (ref = yes)							**1.19**	**1.06–1.34**

Bold value indicates significant associations at *p* < 0.05

## Discussion

Our findings indicate that mental health problems increased among Swedish adolescents between 2014 and 2020, while the prevalence of bullying at school remained stable (< 1% increase), except among girls in year 11, where the prevalence increased by 2.5%. As previously reported [5, 6], mental health problems were more common among girls and older adolescents. These findings align with previous studies showing that adolescents who are bullied at school are more likely to experience mental health problems compared to those who are not bullied [3, 4, 9]. This detrimental relationship was observed after adjustment for school-related factors shown to be associated with adolescent mental health [10].

A novel finding was that boys who had been bullied at school reported a four-times higher prevalence of mental health problems compared to non-bullied boys. The corresponding figure for girls was 2.5 times higher for those who were bullied compared to non-bullied girls, which could indicate that boys are more vulnerable to the deleterious effects of bullying than girls. Alternatively, it may indicate that boys are (on average) bullied more frequently or more intensely than girls, leading to worse mental health. Social support could also play a role; adolescent girls often have stronger social networks than boys and could be more inclined to voice concerns about bullying to significant others, who in turn may offer supports which are protective [21]. Related studies partly confirm this speculative explanation. An Estonian study involving 2048 children and adolescents aged 10–16 years found that, compared to girls, boys who had been bullied were more likely to report severe distress, measured by poor mental health and feelings of hopelessness [22].

Other studies suggest that heritable traits, such as the tendency to internalize problems and having low self-esteem are associated with being a bully-victim [23]. Genetics are understood to explain a large proportion of bullying-related behaviors among adolescents. A study from the Netherlands involving 8215 primary school children found that genetics explained approximately 65% of the risk of being a bully-victim [24]. This proportion was similar for boys and girls. Higher than average body mass index (BMI) is another recognized risk factor [25]. A recent Australian trial involving 13 schools and 1087 students (mean age = 13 years) targeted adolescents with high-risk personality traits (hopelessness, anxiety sensitivity, impulsivity, sensation seeking) to reduce bullying at school; both as victims and perpetrators [26]. There was no significant intervention effect for bullying victimization or perpetration in the total sample. In a secondary analysis, compared to the control schools, intervention school students showed greater reductions in victimization, suicidal ideation, and emotional symptoms. These findings potentially support targeting high-risk personality traits in bullying prevention [26].

The relative stability of bullying at school between 2014 and 2020 suggests that other factors may better explain the increase in mental health problems seen here. Many factors could be contributing to these changes, including the increasingly competitive labour market, higher demands for education, and the rapid expansion of social media [19, 27, 28]. A recent Swedish study involving 29,199 students aged between 11 and 16 years found that the effects of school stress on psychosomatic symptoms have become stronger over time (1993–2017) and have increased more among girls than among boys [10]. Research is needed examining possible gender differences in perceived school stress and how these differences moderate associations between bullying and mental health.

### Strengths and limitations

Strengths of the current study include the large participant sample from diverse schools; public and private, theoretical and practical orientations. The survey included items measuring diverse aspects of the school environment; factors previously linked to adolescent mental health but rarely included as covariates in studies of bullying and mental health. Some limitations are also acknowledged. These data are cross-sectional which means that the direction of the associations cannot be determined. Moreover, all the variables measured were self-reported. Previous studies indicate that students tend to under-report bullying and mental health problems [29]; thus, our results may underestimate the prevalence of these behaviors.

In conclusion, consistent with our stated hypotheses, we observed an increase in self-reported mental health problems among Swedish adolescents, and a detrimental association between bullying at school and mental health problems. Although bullying at school does not appear to be the primary explanation for these changes, bullying was detrimentally associated with mental health after adjustment for relevant demographic, socio-economic, and school-related factors, confirming our third hypothesis. The finding that boys are potentially more vulnerable than girls to the deleterious effects of bullying should be replicated in future studies, and the mechanisms investigated. Future studies should examine the longitudinal association between bullying and mental health, including which factors mediate/moderate this relationship. Epigenetic studies are also required to better understand the complex interaction between environmental and biological risk factors for adolescent mental health [24].

## Supplementary Information

**Additional file 1.** Principal factor analysis description.

## Data Availability

Data requests will be considered on a case-by-case basis; please email the corresponding author.
