# Supplementary material for: Bullying at school and mental health problems among adolescents: a repeated cross-sectional study
Source: Child Adolesc Psychiatry Ment Health. 2021 Dec 14;15:74. doi: 10.1186/s13034-021-00425-y (PMC8672638; doi:10.1186/s13034-021-00425-y)
Supplement: Supplementary file 1 — Additional file 1. Principal factor analysis description. [file 13034_2021_425_MOESM1_ESM.docx]

**Additional file**

**Principal factor analysis description:**

Factor 1, Praise and participation, consists of five statements that measure the degree of praise students receive for good school performance and students' participation in teaching. A high degree of praise and participation is connected to a positive school situation. Factor 2, Interesting and meaningful work, consists of two statements that load negatively on the factor and relates to schoolwork being confusing and meaningless, in addition to two statements that load positively and measure the teaching methods used in school. Items loading negatively were reversed. Schoolwork that is perceived as meaningful and interesting is related to a positive school situation. Factor 3, Rule knowledge and feeling well, consists of three statements about rule knowledge and well-being at school. To know the rules and feel well is associated to a positive school situation. The fourth factor, Poorly structured school lessons, consists of two statements about the perceived structure and tidiness of school lessons. Both items were reversed so that better organized lessons related to a positive school situation. Factor 5, Teachers give no praise for good achievements, consists of a single statement that teachers do not praise good school performance. This item was also reversed to be in concordance with a positive school situation. A total score was calculated for each factor and dichotomised using the median-split.
